# Editorial: New insights into dyserythropoiesis: from pathophysiology, molecular mechanisms to treatments for erythroid disorders

**DOI:** 10.3389/fcell.2023.1321170

**Published:** 2023-11-02

**Authors:** Shujing Zhang, Yang Mei, Baobing Zhao

**Affiliations:** ^1^ Key Laboratory of Chemical Biology, School of Pharmaceutical Sciences, Cheeloo College of Medicine, Ministry of Education, Shandong University, Jinan, China; ^2^ NMPA Key Laboratory for Technology Research and Evaluation of Drug Products, School of Pharmaceutical Sciences, Cheeloo College of Medicine, Ministry of Education, Shandong University, Jinan, Shandong, China; ^3^ Department of Pharmacology, School of Pharmaceutical Sciences, Cheeloo College of Medicine, Ministry of Education, Shandong University, Jinan, China; ^4^ Key Laboratory of Medical Virology, School of Biomedical Sciences, Hunan University, Changsha, China

**Keywords:** erythropiesis, erythroid disorder, erythropoietin, red blood cell, hypoxia

Erythropoiesis is a highly regulated, multistep process in which hematopoietic stem cells differentiate into mature enucleated red blood cells (RBCs), which can be divided into 3 stages: early erythropoiesis, terminal erythroid differentiation, and reticulocyte maturation (Hattangadi et al., 2011; Dzierzak and Philipsen, 2013). Early erythropoiesis starts with the commitment of multi-lineage progenitors into erythroid progenitor cells, followed by the proliferation and differentiation into erythroid burst-forming unit cells (BFU-Es) and subsequent erythroid colony-forming unit cells (CFU-Es). Differentiation from CFU-Es to mature red blood cells, termed terminal erythropoiesis, involves a series of steps including proerythroblasts, basophilic erythroblasts, polychromatic erythroblasts, and orthochromatic erythroblasts that enucleate to become reticulocytes (Zhao et al., 2016). The nascent reticulocyte undergoes further maturation through membrane and proteome remodeling and organelle clearance to become mature red blood cells (Mei et al., 2020).

Red blood cells (RBCs) produced *in vitro* have the potential to alleviate the worldwide demand for blood transfusion (Dias et al., 2011; Dolgin, 2017). A better understanding of the cellular and molecular basis of erythropoiesis, will provide cues and potential targets for the RBCs produced *in vitro.* In this Research Topic, Zhang et al. identified Transglutaminase 2 (TGM2) as a key regulator in the primary fetal liver erythroid differentiation via its cross-linking enzyme activity. TGM2 is a versatile enzyme that modulates cell survival and differentiation via multiple enzymatic activities including cross-linking, guanosine 5′-triphosphate (GTP) hydrolysis, scaffolding, protein disulfide isomerization, serotonylation and protein kinase activity (Eck et al., 2014). Zhang et al. found that TGM2 is upregulated during terminal erythroid differentiation. Its cross-linking activity inhibition but not inhibition disrupted the erythroid maturation and enucleation, which is evidenced by the arrested differentiation at basophilic erythroblast stage and interfered with cell cycle progression.

Erythropoietin (EPO), produced in the kidney in a hypoxia responsive manner, is required for erythroid differentiation. In non-erythroid tissue, EPO increases the production of nitric oxide (NO) via endothelial nitric oxide synthase (eNOS), which contributes to EPO cardioprotective activity in mouse models (Mihov et al., 2009; Teng et al., 2011). However, the role of EPO-induced NO production in erythroid cells is totally unknown. Lee et al. revealed that EPO activated neuronal nitric oxide synthase (nNOS) but not eNOS in erythroid cells, which is required for normal erythropoietic response. This is evidenced by the reduced hematocrit in nNOS knockout mice (nNOS−/−) compared to the equal hematocrit in WT and eNOS−/−mice. In line with this, nNOS inhibition resulted in decreased EPO-dependent proliferation mediated in part by decreased erythropoietin receptor expression, and decreased proliferation of erythroid cells. These data provide evidence that NO modulates EPO-dependent erythropoietic response.

Erythropoiesis is also triggered by numerous cellular physiological processes, including low oxygen concentration (<5%). Hypoxia inducible factors (HIFs) have been proven to promote erythropoiesis via the upregulation of EPO production (Anderson et al., 2011; Rankin et al., 2012; Franke et al., 2013). Gao et al. revealed HIF-2α and insulin receptor substrate 2 (IRS2) mediated the progression of erythroid differentiation under hypoxia. IRS2 acted as a downstream effector of HIF-2α to regulate the erythroid differentiation, providing a novel targeting for promoting erythroid differentiation.

Erythroid disorders, in which abnormal erythrocyte maturation and/or morphology is associated with ineffective erythropoiesis, can result from direct impairment of medullary erythropoiesis (e.g., thalassemia syndromes), inherited bone marrow failure (e.g., Myelodysplastic Syndrome), red cell overproduction (e.g., Polycythemia Vera), or existing blood cell destruction (e.g., Sickle Cell Anemia). It can also be caused by immune-mediated RBC destruction or certain genetic diseases that predispose to abnormal RBC production or turnover (Zhao et al., 2018). Identification the key genes that are involved in the pathogenesis of these erythroid disorders as well as other disease driven by similar mechanisms, provides insights into the new therapeutic modalities for the disease treatment. Acute lymphoblastic leukemia (ALL) is a malignant disease of abnormal proliferation of lymphocytes in bone marrow. Chen.et al. evaluated a potential prognostic signature with six genes and constructed a risk model significantly closely related to ALL. These findings may help clinicians adjust treatment plans to differentiate patients with good and poor prognosis for targeted treatment.
